# bertha: Project skeleton for scientific software

**DOI:** 10.1371/journal.pone.0230557

**Published:** 2020-03-23

**Authors:** Michael Riesch, Tien Dat Nguyen, Christian Jirauschek

**Affiliations:** Department of Electrical and Computer Engineering, Technical University of Munich, Munich, Germany; National Library of Medicine, UNITED STATES

## Abstract

Science depends heavily on reliable and easy-to-use software packages, such as mathematical libraries or data analysis tools. Developing such packages requires a lot of effort, which is too often avoided due to the lack of funding or recognition. In order to reduce the efforts required to create sustainable software packages, we present a project skeleton that ensures the best software engineering practices from the start of a project, or serves as reference for existing projects.

## 1 Introduction

In a recent essay in Nature [1], a familiar dilemma in science was addressed. On the one hand, science relies heavily on open-source software packages, such as libraries for mathematical operations, implementations of numerical methods, or data analysis tools. As a consequence, those software packages need to work reliably and should be easy to use. On the other hand, scientific software is notoriously underfunded and the required efforts are achieved as side projects or by the scientists working in their spare time.

Indeed, a lot of effort has to be invested beyond the work on the actual implementation—which is typically a formidable challenge on its own. This becomes apparent from literature on software engineering in general (such as the influential “Pragmatic Programmer” [2]), and in scientific contexts in particular (e.g., [3–6]). The vast number of best practices guides and development guidelines available (e.g., those published by the German Aerospace Center (DLR) [7] and the Netherlands eScience Center [8]) further underlines the importance of the topic and may serve as guidance, but often scientists lack the time and/or formal training in software engineering required to ensure sustainable software development [1, 5, 6]. Too often, this results in poorly maintained software projects of questionable reliability and usability.

Given all this, once again the goal is to achieve much with little effort. Therefore, in this paper we present a project skeleton that may serve as solid yet lightweight base for a small to medium-scale scientific software project. In the use case we envisage, scientists can create an instance of this template in just a few clicks. This instance implements essential best practices in software engineering from the very start. After performing a minimal number of customizations, the scientist can soon start working on the actual implementation and can concentrate on what really matters. In the context of pure C++ [9] or Python [10] projects, such skeletons have already proven their value. In the scope of this work, we focus on scientific software libraries which are written in the C++ programming language for performance reasons and feature bindings for Python in order to provide an easy-to-use interface to the user. These programming languages are widely accepted in both open-source and high-performance computing (HPC) communities, and should therefore be considered a reasonable choice. To the best of our knowledge, a project skeleton for this combination of programming languages has not yet been published.

It should be noted that the skeleton does not (and should not) cover every eventuality (e.g., when support for the Fortran programming language is required) but concentrates on one particular use case. This is contrary to the recommendations in related literature, which are kept general and language-agnostic on purpose. The rationale behind this decision is to keep the template lightweight and avoid cluttering.

The paper at hand is organized as follows: In Section 2, we identify the essential best practices that are required to ensure high-quality scientific software based on related literature and our own experiences with our software projects (e.g., the mbsolve software [11, 12], a solver for the generalized Maxwell-Bloch equations [13]). Subsequently, we present our project skeleton and discuss the specific implementation of the measures identified in Section 3. As already stated above, some minor customization steps are required. Section 4 gives an overview of these steps and thereby an introduction to the (potential) user. Finally, we conclude with a short summary and give an outlook on future work, i.e., additional tools and measures that further improve the quality of scientific software projects.

## 2 Best practices in scientific software engineering

This section describes the essential recommendations and best practices from related literature [1–8] that serve as basis for the project skeleton. All recommendations are language-agnostic and grouped into seven categories with no particular order of importance. Table 1 gives an overview of the best practices.

**Table 1:** Overview of best practices in software engineering for scientific software projects. For each best practice, implementation candidates are listed where the selected choice is denoted in bold.

Group	Best practice	Implementation candidates (not exhaustive)
Project management	Version control system	**git**, mercurial, svn
	Project management tool	**GitLab**, **GitHub**, Bitbucket, JIRA
	Workflow	**GitLab Flow**, GitHub Flow, git flow
Coding style	Code formatting style	**Mozilla**, LLVM, Google, Chromium
	Code formatting tool	**clang-format**
	Static code analysis	clang-tidy, cppcheck, cpplint
Independence	Use open file formats	e.g., JSON, CSV, HDF5
	Use open-source libraries	e.g., Eigen, FFTW, GNU Scientific Library
Automation	Continuous integration	**gitlab-ci**, **Travis CI**, AppVeyor, Microsoft Azure
	Build automation	**CMake**, GNU make, Bazel, Ninja, MS Build
Documentation	Function reference	**Doxygen**, Sphinx (with Breathe)
	“Big picture” documentation	**Markdown**, reStructuredText
Testing	Unit test framework	**Catch2**, Google Test, Boost Test Library
	Code coverage report	**gcov**, various commercial tools
Deployment	Package binaries	**conda**, Conan, Debian apt
	Online documentation	**GitLab Pages**, **GitHub Pages**, readthedocs.io

### 2.1 Project management

Most software projects in a scientific context start with a single developer. However, over time the projects are likely to grow, be extended, and possibly taken over by other developers. Building a developer community is crucial for the success of the project in general and in particular for open-source projects [4]. Therefore, the project infrastructure should be able to handle multiple developers from the very start.

All of the guidelines in literature we have found mention the usage of a version control system (VCS). This is beneficial even for the single developer, as a VCS intrinsically features a backup solution and synchronization between different machines. Once more developers start working on the project, this enables transparent collaboration. By using a VCS, the “Make Incremental Changes” paradigm [5, 6] can be implemented easily and the intrinsically generated development history may serve as rudimentary documentation of design decisions [4].

In a more advanced scenario, the VCS is coupled with a project management tool that provides a means of communication within the developer team, and thereby further enhances transparency. As the communication logs are available for developers who join the team at a later stage, this also provides a certain form of documentation [4]. One essential element of a project management tool is a ticket system or issue tracker. Issues are requests for a certain change (such as a bug fix or feature implementation) and play a crucial role in modern iterative and incremental software development processes, such as feature-based development [14]. As the name suggests, issue trackers keep track of issues from their creation (by users or developers) to their completion in the form of an accepted solution by the developer [7]. Modern project management tools also include convenient mechanisms for code review. Similar to a scientific paper, a rigorous review process may be time-intensive and annoying, but eventually yields solutions of higher quality and wider acceptance [5].

### 2.2 Code quality

Just as we care about language style when writing a scientific article, so we should care about coding style when writing scientific software. Here, we should bear the mottos “Write Programs for People, Not Computers” [5] and “Don’t Repeat Yourself” [2, 5] in mind and produce easily readable and modular code. In developer teams, it is crucial to agree on a certain coding style at the beginning of the project. The coding style usually consists of two parts: rules for formatting code and best practices for programming in the respective language. Code formatting tools enable manual and automated checks to establish whether the source code is compliant with agreed code formatting rules [8]. Analogously, static code analysis tools check whether the agreed best practices are violated [7].

### 2.3 Independence

Some guidelines recommend that open standards, protocols, and file formats should be used wherever possible (e.g., the HDF5 format for large data sets [8]). Thereby, vendor lock-in situations are avoided which would arise, for example, if a certain source code can only be compiled using a certain compiler brand or version. Our general recommendation here is to provide solutions that work with the most widely used operating systems and compilers (and possibly combinations thereof) from the very start.

Following the advice that one should never reinvent the wheel, established software libraries and tools are often used to speed up development processes. Here, we recommend using open-source components unless there is a strong reason not to. This is in agreement with the interoperability and reusability part of the FAIR principle [15, 16].

### 2.4 Automation

We should “Let the Computer Do the Work” [2, 5] and automate repetitive tasks such as building the software, running tests, performing quality checks, and deploying the generated artifacts (typically, software in binary form and documentation) to a software repository. Otherwise, those tedious tasks are most likely postponed, not done at all, or performed only partially. Here, continuous integration (CI) tools are helpful as different jobs can be defined and grouped into stages, which are executed every time the developers push changes to the version control repository. Then, the developers receive feedback on the changes, which is an essential part of the “Make Incremental Changes” strategy [5].

The feedback typically consists of (at least) two parts, which are briefly outlined. First, the build process should run in an automated and platform-independent fashion. Here, it is particularly important that third-party dependencies are found without hard coded paths. The output of the build process tells the developers whether the build on different platforms was successful. This is especially beneficial as most developers develop on a certain platform and the code is not intrinsically tested on other platforms (different operating systems, different compiler versions, etc.). Second, test programs can be executed automatically on different platforms. For example, unit tests can help to verify the correct behavior of certain functions or modules of the software. Functional tests, on the other hand, help to gain more confidence in the overall function of the software [7].

It makes sense to define the continuous integration pipelines as early as possible, so that the developers benefit from the feedback from the very beginning. Thereby, bugs in the software (in particular regressions) can be detected early. Furthermore, the effectiveness of optimizations can be assessed while the correct operation of the software is ensured.

### 2.5 Documentation

In order to make scientific software reusable, providing documentation to users and developers is one of the most important steps [1–8]. Bangerth and Heister [4] list five items that the documentation should contain: traditional comments, function level documentation, class level documentation, overview of how modules interact, and complete examples in tutorial form. As to traditional comments, it is good practice to “Document Design and Purpose, Not Mechanics” [5] and avoid obvious comments. Function and class level documentation is typically generated based on comments in code using special annotation. The resulting reference manual is particularly interesting for developers and advanced users who need to know the details. On the other hand, the module overview documentation should inform new users about the big picture. This information is typically written into the files README (aim of the software, installation notes, list of dependencies), CHANGELOG (overview of releases, features, known bugs), CODE_OF_CONDUCT and CONTRIBUTING (guidelines for (potential) developers), as well as TUTORIAL (guide for (potential) users) [6].

### 2.6 Testing

As mistakes are natural and are bound to happen, we should plan for them and develop strategies on how to detect them as early as possible [5]. Automated testing, the importance of which has already been underlined in Section 2.4, is the cornerstone of such strategies. It should be noted that the effectiveness of tests should be monitored as well. Here, code coverage tools are useful as they are able to detect code parts which are not covered by the executed tests [7].

Again, we stress that certain measures, such as writing unit tests, should be carried out from the very beginning. Apart from their use in automated testing, unit tests may have a positive effect on the code design. Since modular code is usually testable, performing unit tests can be considered a necessary requirement for modular code [2].

### 2.7 Deployment

Whether or not a certain software project is used depends to a large degree on the ability to distribute it [4]. Hence, it is advisable to package the software and distribute it using an established software repository [1]. Similar to the practices discussed above, it is important that the deployment is carried out automatically and as early as possible [3].

## 3 Implementation of the project skeleton

**Fig 1:**
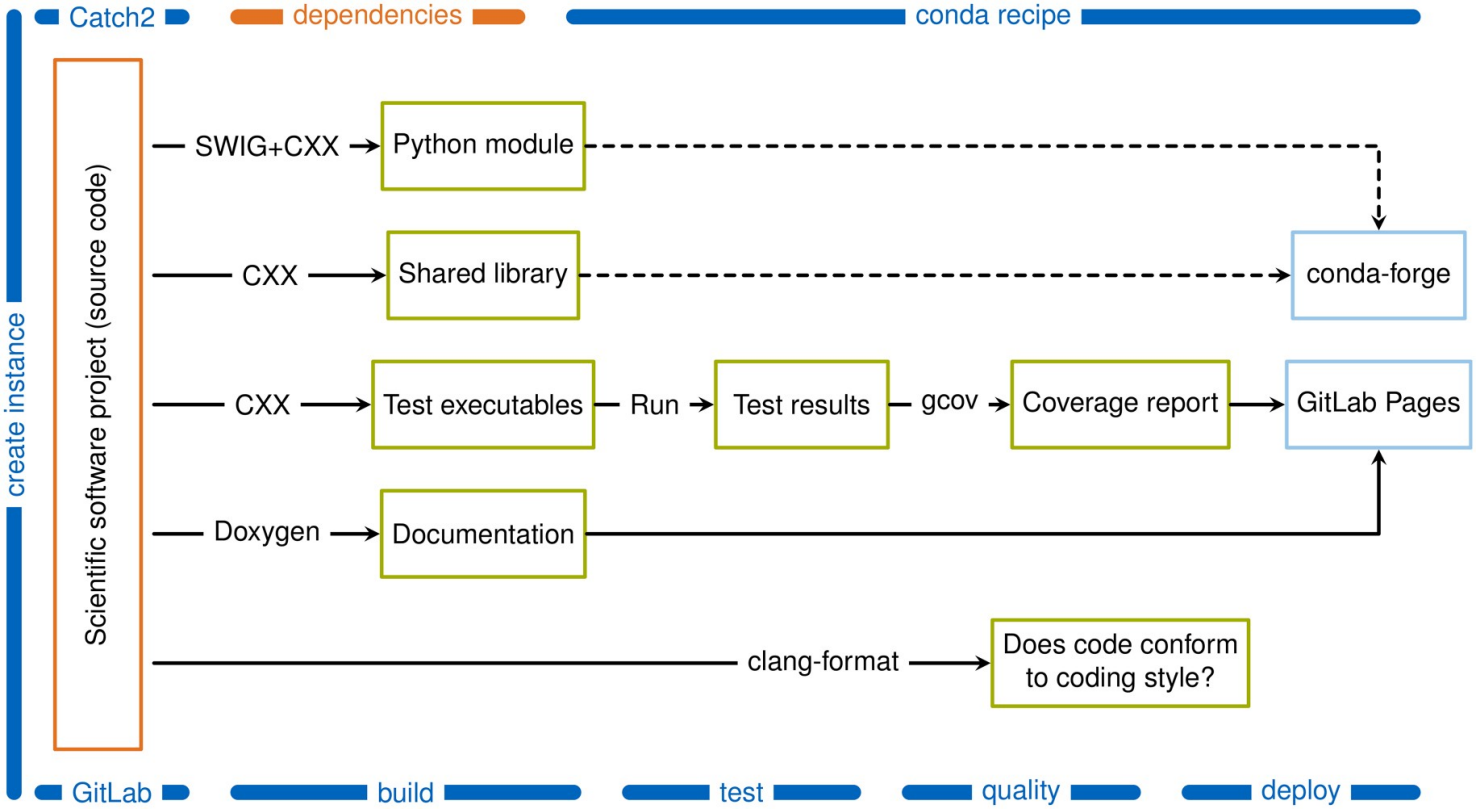
Overview of the project skeleton. The source code and dependencies of a scientific software project are denoted in orange. These are the parts the developer has to provide. The presented skeleton guides the project from creation to deployment. Here, the arrows denote jobs that are created by the CMake build system. These jobs are triggered during the different continuous integration stages (build, tests, quality, deploy) or (in the case of the dashed arrows) by the conda-forge build service that follows the recipe [18]. The job names indicate the tools in use, where CXX represents one of the C++ compilers that are supported by CMake.

Based on the (general and language-agnostic) best practices introduced in the section above, we implement measures for a C++ software library with bindings for the Python language in this section. The result is publicly available [17] and may serve as a template for new projects or reference for existing projects. Fig 1 outlines the skeleton approach.

It should be noted that there may be different ways to implement a certain measure. For the sake of simplicity, we discuss only one or two possibilities for most measures. Following the recommendations in Section 2.3, we have selected open-source tools and libraries exclusively. Thereby, one particular lightweight solution is provided for scientists who are new to the topic, while the advanced users may replace a certain implementation of a measure with another library or tool of their choice.

Since a project skeleton does not include a real implementation, best practices regarding planning, structuring, and writing code can hardly be demonstrated. In this regard, we refer the reader to available literature on the topic, such as [2], and focus on the project skeleton that provides the required infrastructure.

### 3.1 Usage of a version control system (VCS) and appropriate workflow

A multitude of version control systems has been published and used over the last three decades. We stick to our criterion that the software must be open-source and note that git has received much attention since it was first released in 2005. It features distributed version control and a flexible branching model, rendering it perfectly suited for open-source projects. However, the flexible branching model might, at the same time, be a significant drawback. Each project should define a workflow to show how changes are developed, tested, and integrated. As usual, it makes sense to use something established, such as the GitLab Flow [19]. This workflow uses feature branches to develop and test new features or bug fixes. Once the changes on the feature branch fulfill the requirements and pass the automated tests and quality checks, the developer can open a merge request. A maintainer can subsequently merge the changes in the main development branch. Additionally, the GitLab Flow allows stable branches and different environments (such as production) in which further restrictions may apply. The latter features are not required at the initial stage of a project, but underline that the GitLab Flow is simple enough for small projects yet powerful enough for large and established projects.

### 3.2 Usage of a project management tool including issue tracking

There are several management tools and hosting platforms that can be combined with the git version control system with different strengths and drawbacks. Here, we would like to leave the choice to the developers and provide two possible solutions for the undecided.

Over the last decade, the GitHub platform has received significant attention. It provides free public git repositories and integrations with other services (such as the zenodo repository for storing research output). Due to its prominence, we have decided to provide a mirror repository of the project skeleton in GitHub [20]. This repository is marked as project template, which allows a new project to be instantiated with a few clicks. As to continuous integration, GitHub offers support for external CI providers such as Travis CI, AppVeyor, and Microsoft Azure. These services are typically free for open-source projects and configured using a YAML file, where CI jobs can be described. As an example, we have added a basic configuration file for Travis CI that triggers build and unit tests on Linux, Windows, and macOS platforms given that the user registers on Travis CI, where the CI operation can be activated for the repository in question.

Alternatively, the GitLab platform can be used, which is conceptually similar to GitHub, the main difference being the possibility of self-hosting the platform on a local server for free. While the concepts (such as Pages and Releases) are similar, there are slight differences. For example, the project template instantiation mechanism is different. At this point, it is not possible to create an instance of the project skeleton with a single click. However, we aim to provide that feature in the near future [21].

GitLab.com provides free hosting and internal continuous integration services for open-source projects. Currently, those internal CI services are restricted to the Linux operating system. It is possible, however, to install GitLab’s CI suite on a local machine and connect it to GitLab. Alternatively, an external service can be used for Windows or macOS operating systems. In the event that the project should not be open-source, the self-hosted operation mode may be selected. Here, the CI suite must be installed on local machines, which can subsequently be connected to the local GitLab installation.

It should be noted that we did not add configuration files for all options to the template in order to provide a lightweight skeleton. Instead, we included the configuration file for the GitLab internal CI, which calls the targets generated by the build systems. From this configuration file, corresponding files for other CI services can be derived.

### 3.3 Automated build system

In particular when the C++ programming language is involved, the CMake project provides well-established tools to build, test, and package software. The main advantage of CMake (compared to alternatives such as GNU make, Visual Studio, or Eclipse) is that a level of abstraction is introduced. The configuration files consist of directives such as add_library or find_package and are, therefore, quite easy to read and understand. Based on those configuration files, project files for the aforementioned alternatives (and many other build systems) can be generated. Thereby, the software project can be built for different operating systems or using different compilers. In addition, CMake features a mechanism for finding third-party libraries and tools. This feature is essential for cross-platform dependency management.

As a proof of concept, we have added a simple shared library written in C++ to our project skeleton. It features a simple class device with two member variables that represent its start and end coordinates, respectively. An instance of this class can be created using one of two constructors, where either the coordinates are specified directly, or the length can be set and the start coordinate is assumed to be at the origin. Finally, a method returns the length of the device.

For such a shared library, Python bindings can be generated conveniently using the SWIG project. It is fully supported by CMake and requires only a minimal configuration file, which basically specifies which C++ header files should be considered when the interface is created. SWIG scans the specified header files and automatically generates a Python module, which can be subsequently imported and used in a Python project.

### 3.4 Unit testing

Ideally, the software is designed so that each unit of software (e.g., a function) fulfills a certain, unique task (“Design by Contract” technique [2]). Furthermore, the implementation of each unit is flawless. While the first goal can be achieved by careful design and refactoring, the second statement is rarely true. As mentioned above, mistakes will happen and we have to test whether the implementations of each unit work correctly.

In the case of our simple C++ library, we have to check, for instance, whether the calculation of the length yields the correct result. This can be achieved by writing a unit test that creates an instance of the device class, calls its get_length method, and compares the result of the method to the expected value. Also, whenever the user specifies input data, the implementation should check whether those values are reasonable and deal with invalid values (most likely, by throwing an exception). Error handling code must be tested as well, for example by creating a unit test in which the error is provoked on purpose and checking whether the error handling code yields the correct behavior. As the number of unit tests is expected to be large for a real life project, it is recommended to use a unit test framework.

We chose the Catch2 library as it is open-source, lightweight and header-only. Based on this library, we added a test executable with several unit tests to our CMake build system. Here, we could rely on the CTest functionality of the CMake project. Whether or not the unit tests cover all possible situations can be assessed using code coverage tools. We have added the possibility of using the gcov tool to the project skeleton. This tool generates profiling information during the execution of tests. This information can be subsequently converted to a human-readable report, in which metrics such as line coverage are given on a per-file basis.

### 3.5 Automatic code formatting

Here, the clang-format tool constitutes a helpful and versatile instrument. It can be configured using a single file, in which the code formatting rules are specified. There are several predefined styles that can be used as-is, or alternatively serve as a basis. It is also possible to define a certain style from scratch, but we recommend using an existent style (with slight modifications, if required).

In our project skeleton, the clang-format tool is integrated into the CMake build system, making it easy for the user to format all source files automatically. This functionality is also used to check whether the source code conforms to the specified style in the scope of continuous integration.

### 3.6 Documentation generation

From the implementation point of view, we can separate the different types of documentation listed in Section 2.5 into two groups, namely the function reference and the overview documentation. The function reference is based on comments in the source code that use special annotation. The information in those comments can be extracted using the Doxygen tool. For the overview documentation, which provides the “big picture”, it makes sense to use a structured text format. Since Doxygen supports the Markdown language, we chose to write files such as README.md and CONTRIBUTING.md in this annotation. Both the overview documentation and function reference are then transformed into static HTML pages that can be viewed locally or uploaded to a web server.

We note that while Doxygen provides unchallenged support for in-source C++ documentation, the design of the generated HTML files appears a bit dated. More advanced workflows are available that use Doxygen as input parser and alternative tools to generate the static HTML pages. However, this is beyond the scope of the work at hand.

### 3.7 Automated packaging and deployment to a public repository

While many operating systems or programming languages feature a common repository for exchanging programs and libraries in binary form, it would be beneficial to have a language-agnostic repository that covers all operating systems. Fortunately, the conda system provides exactly this. Once a software project is in a stable state, a recipe can be created on conda-forge that defines the source of the project, the steps required to build it, and meta information such as the name of the responsible maintainer. Based on this recipe, the conda-forge build system automatically generates the binaries for different platforms. Then, on each platform the resulting package can be easily installed within a conda environment.

Most likely, the package has dependencies on other libraries. The conda system offers a vast number of third-party components and convenient methods of installing them. The environment approach already mentioned has a positive effect on the dependency management, as in Windows it is generally impossible to distinguish between different versions of a library (at least when considering unmanaged C++ code), dubbed the “DLL Hell”. Using conda environments, however, it is possible to separate different versions in a clean and convenient way.

The documentation generated could be included in a conda package as well. However, we found it more appropriate to publish it on a web server for visibility reasons. Both GitHub and GitLab offer the possibility of hosting static HTML pages, such as those generated by Doxygen. With a few lines of CI configuration, the documentation is automatically generated and uploaded. See [22] for an example.

## 4 Creating a skeleton instance

**Fig 2:**
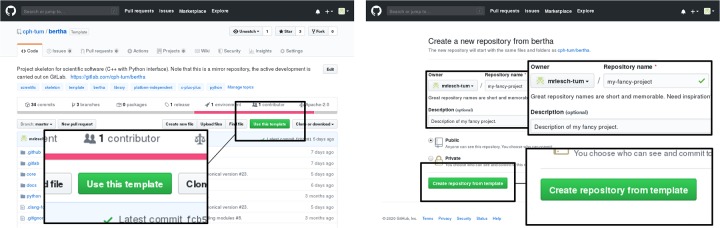
Creating an instance of the project skeleton on GitHub. On the project page of bertha [20], click on “Use this template”. In the following, enter the desired owner, repository name, and project description. The button “Create repository from template” will then create the instance.

**Fig 3:**
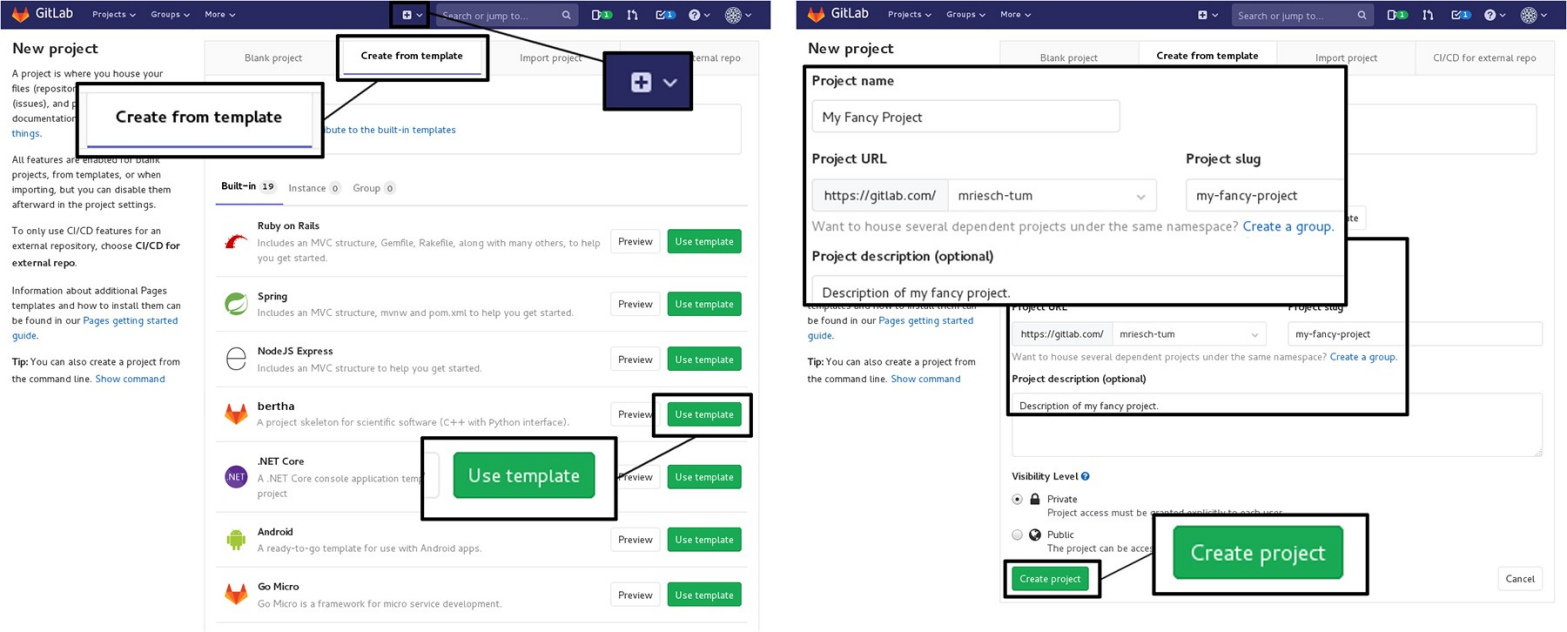
Creating an instance of the project skeleton in GitLab. Click on the plus button to create a new project. After selecting the “Create from template” tab, choose bertha by clicking “Use this template” (currently in development, see [21]). Then, enter the project name and description and click “Create project”.

In order to create a new project, the project skeleton can be cloned using the mechanisms of either GitHub (see Fig 2) or GitLab (as described in Fig 3). Alternatively, the files can be copied manually and added to a new repository. After the cloning procedure, the skeleton can be adjusted to the needs of the new project. The steps recommended and required are outlined briefly in the following. For more detailed instructions, please refer to the “Tutorial” section in the bertha documentation [22]. Please note that registration on Travis CI and activation of the project is required for continuous integration support in GitHub.

### 4.1 Setup stage

At the beginning, it is important to define a meaningful name for the project and replace bertha with this name throughout the project (e.g., in the CMake build structure). Ensure that the name is not already used (e.g., in conda-forge) if the project is to be open-source. Then, the project team should agree on where to host the project (for internal use only or publicly available), on the license for the project, and on the workflow. The latter includes mainly the coding style and the version control workflow. Both should be documented as soon as possible.

### 4.2 Implementation stage

At this point, the software project has a solid initial state. Now it is time to add functionality. Here, consider writing the documentation first (the contract), then implementing the functionality, and at the same time writing unit tests. This approach will seem slow but improves the quality of the design and helps to detect mistakes early on. Also, the CMake build structure can be adjusted to add requirements (e.g., software libraries) or additional modules (besides the existing core library).

### 4.3 Publication stage

In the case of an open-source project, the code should be distributed and communicated as soon as it has some first functionality. For the distribution of the project in binary form, the conda recipe for bertha [18] may serve as reference.

## 5 Conclusion

In the work at hand, we have presented a skeleton for scientific software projects which consist of libraries written in the C++ programming language and feature a Python interface. The skeleton contains the essential elements required to ensure best software engineering practices. With this, we hope to provide the scientific community with a helpful tool that saves time during the setup of a new project. Based on our experience gained during the development of the skeleton, creating a bertha instance may replace at least one person month of evaluating tools, reading documentation, and searching for answers in the internet.

Furthermore, this contribution may serve as checklist and reference for existing projects. We hope that in both use cases—building a project from scratch and adapting an existing one—the project skeleton will aid the implementation of good practices in scientific software engineering and consequently improve the quality and reusability of scientific software projects. At the moment, two of our simulation software projects are based on bertha. The mbsolve software mentioned above is an open-source project in which bertha is used to adapt the existing project. On the other hand, we have created the first instance of bertha for a solver tool that is dedicated to the numerical modeling of rapidly wavelength-swept Fourier domain mode-locked fiber lasers [23, 24]. This tool is developed in-house, which is allowed by the permissive license of the bertha project. As we are going to use bertha in further simulation projects, we plan to maintain it in the future. Nevertheless, we hope that in the near future a community will form around the project skeleton that will use, maintain, and extend bertha.

As a next step, the implementation of further measures is envisaged. For example, a static code analysis tool could further improve the quality of the code. Also, the generated documentation and quality reports should be presented with a modern appearance. Finally, the project skeleton concept can be transformed to other project classes in scientific software engineering, such as a combination of a Fortran library with a Python interface.
